# “I Wasted 3 Years, Thinking It’s Not a Problem”: Patient and Health System Delays in Diagnosis of Leprosy in India: A Mixed-Methods Study

**DOI:** 10.1371/journal.pntd.0005192

**Published:** 2017-01-12

**Authors:** Thirumugam Muthuvel, Srinivas Govindarajulu, Petros Isaakidis, Hemant Deepak Shewade, Vasudev Rokade, Rajbir Singh, Sanjeev Kamble

**Affiliations:** 1 German Leprosy and TB Relief Association, Chennai, India; 2 Médecins Sans Frontières (MSF)/Doctors Without Borders, Mumbai, India; 3 International Union Against Tuberculosis and Lung Disease (The Union), South-East Asia Office, New Delhi, India; 4 Assistant Director of Health Services (Leprosy), Government of Maharashtra, Pune, India; 5 State Leprosy Officer/Joint Director of Health Services (Leprosy), Government of Maharashtra, Pune, India; Fondation Raoul Follereau, FRANCE

## Abstract

**Background:**

Worldwide, leprosy is one of the major causes of preventable disability. India contributes to 60% of global leprosy burden. With increasing numbers of leprosy with grade 2 disability (visible disability) at diagnosis, we aimed to determine risk factors associated with grade 2 disability among new cases and explore patients and providers’ perspectives into reasons for late presentation.

**Methodology/Principal Findings:**

This was an explanatory mixed-methods study where the quantitative component, a matched case-control design, was followed by a qualitative component. A total of 70 cases (grade 2 disability) and 140 controls (grade 0) matched for age and sex were randomly sampled from new patients registered between January 2013-January 2015 in three districts of Maharashtra (Mumbai, Thane and Amaravati) and interviewed using a structured close ended questionnaire. Eight public health care providers involved in leprosy care and 7 leprosy patients were purposively selected (maximum variation sampling) and interviewed using a structured open-ended interview schedule. Among cases, overall median (IQR) diagnosis delay in months was 17.9(7–30); patient and health system delay was 7(4–16.5) and 5.5(0.9–12.5) respectively; this was significantly higher than the delay in controls. Reasons for delayed presentation identified by the quantitative and qualitative data were: poor awareness of leprosy symptoms, first health care provider visited being private practitioners who were not aware about provision of free leprosy treatment at public health care facilities, reduced engagement and capacity of the general health care system in leprosy control.

**Conclusions:**

Raising awareness in communities and health care providers regarding early leprosy symptoms, engagement of private health care provider in early leprosy diagnosis and increasing capacity of general health system staff, especially targeting high endemic areas that are hotspots for leprosy transmission may help in reducing diagnosis delays.

## Author Summary

Leprosy is caused by bacteria that affects nerves, causing deformities. These deformities are preventable through early detection and prompt treatment. India reports more than 60% of world’s leprosy burden, of which new case with deformities is at increasing trend in the recent years, which is a public health issue. However, long delay in diagnosis have reported. In this study, we observed that patient delay is larger than health care provider delay. Many patients were ignorant of early symptoms of leprosy. In addition, we observed that limited community activities and misdiagnosis appears to be contributing to overall delay in diagnosis in areas of Maharashtra, India. Although more research on this subject should be conducted, this study highlights a need for further educational interventions for both patients and training for doctors.

## Introduction

Leprosy is one of the major causes of preventable disability and considered to be important public health problem mainly because of its potential to cause permanent and progressive physical deformities with serious social and economic consequences [1]. Despite global success in the control of leprosy, delayed diagnosis and the resulting grade 2 disabilities (defined as any visible disability like ulcers, claw hand/toe, muscle wasting) remains important public health challenge [2]. The longer the delay, the more is the likelihood of risk of nerve damage with subsequent disability. Therefore, the proportion of grade-2 disability among new cases reflects the level of the disease awareness in the community, as well as the capacity of the health system to detect new cases early.

A total of 213,899 new cases were detected from 102 countries in 2014–15; of which India contributed 125,785 (59%) [3]. In India, the National Leprosy Eradication Programme (NLEP), previously a vertical programme, was integrated into the general health system in 2005. On 30^th^ January 2006, Government of India announced ‘elimination of leprosy as a public health problem’ (prevalence rate <1/10,000) at national level which was actually an achievement of intensified control (something between control and elimination of disease) and not ‘elimination of leprosy as disease’ (zero incidence of leprosy) per se. *“Because in case of leprosy*, *elimination efforts were directed to control the diseases rather than infection*, *by using prevalence instead of incidence of disease”* [4]. The Government of India instructed all the states to “stop all active search for case detection” the same year [5] since they would not be cost effective.

The ‘Enhanced Global Strategy for further reducing the disease burden due to leprosy 2011–2015’ by the World Health Organization targeted a 35% reduction of grade-2 disability among new cases by 2015 as compared to 2011; whereas, in India, there was a 50% increase in grade-2 disability among new cases during the same time period (3% in 2010–11 and 4.6% in 2014) [3].

Minimizing delays in case detection remains an important programmatic challenge, and understanding and addressing the underlying mechanisms may substantially add to the performance of the NLEP, and increasing proportion of grade-2 disability among new cases. Therefore, a large multi-centric study was conducted across five states in India, supported by Indian Council of Medical Research, New Delhi, India [6]; here we present the results for the state of Maharashtra.

A mixed methods study was conducted in Maharashtra, India (2013–15) to understand the current status of the diagnosis pathway including delays and explore the factors affecting the same among newly diagnosed patients with leprosy, both from providers’ perspective and patients’ perspective. ***Specific objectives*** were to i) determine diagnosis delay (patient and health system delay) among those with grade-0 (no disability) and grade-2 disability; ii) determine the factors associated with grade 2 disability; iii) determine the reasons for not visiting a health care provider, including in a public health facility; and iv) explore the patient-provider perspectives into reasons for late presentation.

## Methods

### Ethics approval

Ethical approval was obtained from the Institutional Review Board (IRB) of German Leprosy and TB Relief Association (GLRA), India and Ethics Advisory Group (EAG) of the International Union Against Tuberculosis and Lung Disease (The Union), Paris, France. Informed written consent was obtained from participants. Permission was taken from relevant authorities before implementing the study.

### Study design

This was an explanatory mixed-methods study [9] i.e. the quantitative component of the study, a matched case-control design, which was followed by the descriptive qualitative component, involving one-on-one interviews of patients and public health providers involved in leprosy services. Findings from the former fed into the latter.

### Study Setting

#### General setting

**Fig 1:**
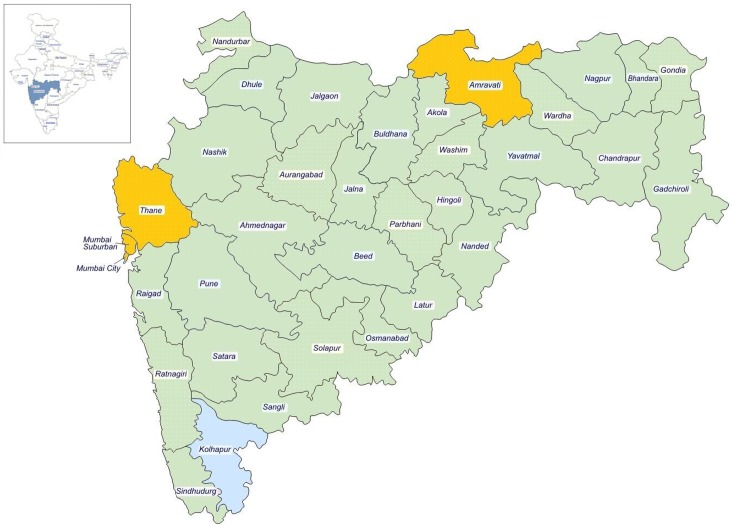
Data collection sites for the study in Maharashtra, India (2013–15): Mumbai, Thane & Palghar and Amaravati.

Maharashtra is a highly populated state in the western region of India with 34 districts and a population of 117 million [7]. Maharashtra is one of the endemic states for leprosy contributing 13% of country’s new case load [8]. A total of 16415 new cases were reported during 2014–15 of which 713 (4.3%) were new cases with grade 2 disability. Three districts were randomly selected for the study using probability proportion to size (PPS) method; Mumbai, Thane and Amaravati. Mumbai is a metropolitan city, while Thane and Amaravati are predominantly rural and tribal areas (Fig 1).

#### Leprosy programme

Under NLEP, there is a district leprosy officers and state leprosy Officer at district and state level respectively. Since the integration of leprosy programme with general health services, suspected cases of leprosy are examined by medical officers posted at Primary Health Centres’ (PHCs’) and they are put on treatment if they are diagnosed to be suffering from leprosy. Those difficult to diagnose cases are referred to District Leprosy Officer for further evaluation. If District Leprosy Officer diagnose them to be suffering from leprosy then they are initiated on treatment and referred to the centre, for continuation of the treatment. Patients’ information is updated at the district leprosy office. Leprosy multi drug treatment kits are made available at the nearest health facility and patients receive their multi drug treatment kit on a monthly basis free of cost.

### Study population

The study population for the quantitative component consisted of newly diagnosed adult (>18 years) patients with leprosy registered for treatment between January 2013 and January 2015. Cases were leprosy patients who reported with grade 2 disability at diagnosis and controls were patients with grade 0 disability. For the qualitative component, the study population included public health care providers involved in leprosy services, and newly diagnosed adult leprosy patients with grade 1 and grade 2 disability.

### Sample size & sampling

For the quantitative component, the sample size was calculated using Epi-info version 3.03.17. Considering 20% prevalence for exposure (poor knowledge of the disease) among control population; Odds Ratio of 2.5; 80% power; 5% alpha error, and the ratio of cases (grade 2) and controls (grade 0) 1:2, the required sample size was 70 cases and 140 controls. Sampling design was a two-stage cluster sampling with equal cluster size. The three study districts Mumbai, Thane and Amravati (primary sampling units), were selected through Probability Proportion to Size (PPS) and the secondary sampling unit were patient with leprosy. A total of 230 grade 2 cases were registered between January 2013 and January 2015 in the three districts of Maharashtra. From the sampling frame, 70 cases and 140 controls were selected after matching for age (± 5 years) and gender. In the situation of not getting consent for participation or non-availability of the selected participant, the next available case or control in the treatment register was enrolled.

For the qualitative component, purposive sampling was done. A total of eight health care providers involved in leprosy services who were knowledgeable and willing to participate and seven patients with leprosy (five with grade 2 disability and two with grade 1 disability) were selected.

### Data collection and variables

For the quantitative component, data for cases and controls were collected between April 2015 and September 2015 using a close-ended structured questionnaire by trained research staff. The variables collected were educational status, occupation, place of residence, knowledge & awareness of the disease, patient delay (duration between notice of 1st symptom to 1st Health Care Provider (HCP) consultation in months), reasons for patient delay, first HCP met, health system delay (duration between 1st HCP and diagnosis in months) and distance from public health centre etc.

For the qualitative component, programmatic factors were explored through one-to-one interviews (structured open ended interview) by the principal investigator who was trained in qualitative research (TM). Results of the quantitative component guided the development of an interview guide which was pilot tested before use. Interviews and analysis were conducted between November 2015 and March 2016. The investigator held interviews at the participants’ workplace. The research objectives were explained to the stakeholders. Prompts were provided to the participant when they were facing difficulty in understanding or hesitating in answering. Audio or video recording was not done as the participants did not consent for the same; the interviewer recorded verbatim field notes during the interview and transcripts were prepared the same day. The summary of the interviews was read back to the interviewees to ensure participant's validation. Interview duration ranged from 10 to 30 minutes. There was no drop out and no repeated interviews were conducted.

### Data analysis

Quantitative data collected were single-entered, validated and analysed using STATA version 12. Mean (SD) and median inter-quartile range (IQR) was used to describe diagnosis delay. Median difference between cases and controls were analysed using Mann-Whitney U test. Unadjusted and adjusted analysis was carried to determine the factors associated with grade 2 leprosy. Variables showing an unadjusted p<0.2 were subjected to conditional logistic regression (method: forward LR). The association between risk factors and grade 2 disability was inferred using adjusted odds ratios (aOR) and 95% confidence interval (95% CI).

Manual descriptive thematic analysis was used to analyze the transcripts by two investigators (TM, PI) [9]. The decision on coding rules and theme generation was done using standard procedures and in consensus [10]. Any difference between the two was resolved by discussion. This approach in health-care research is flexible and appropriate for determining solutions to real-world problems. Similar codes were combined into themes [11]. To ensure that the results are a reflection of the data, the codes/themes were related back to the original data [12]. The findings were reported by using ‘Consolidated Criteria for Reporting Qualitative Research’ [13].

## Results

### Quantitative findings

Table 1 presents the diagnosis delay among cases and controls. Among cases, overall median (IQR) diagnosis delay in months was 17.9 (7–30); Mean ± SD was 23.2±21.9 months. This was significantly higher than the delay in controls (p<0.01)

**Table 1:** Diagnosis delay in months and its association with grade 2 disability at diagnosis among adult new leprosy patients registered in Maharashtra, India (2013–15).

	Cases n = 70	Controls n = 140	Cases n = 70	Controls n = 140	p-value*
Delay (in months)	Median [IQR]	Median [IQR]	Mean ± SD	Mean ± SD	
Patient delay	7(4–16.5)	2(0.5–6.8)	14.8±19.5	7.2±16.7	<0.05
Health system delay	5.5(0.9–12.8)	2 (0.1–5.4)	8.4±9.6	3.9±5.8	<0.05
Total delay	17.9(7–30)	4(2–13.7)	23.2±21.9	11.1±18.9	<0.01

IQR = Interquartile range; SD = Standard Deviation

*Mann Whitney U test

Table 2 presents the risk factors for grade 2 disability among patients with leprosy. Socio-demographic characteristics of cases and controls were comparable. Patient delay (>1 month), first consultation with a private provider and alcohol usage were statistically significant risk factors for grade 2 disability. Majority of the cases (69%) first sought care from qualified private provider after the notice of first symptom. But majority of controls (54%) first sought from public health system.

**Table 2:** Risk factors associated with grade 2 disability at diagnosis among adult new leprosy patients registered in Maharashtra, India (2013–15).

	Cases		Controls		Crude OR (95% CI)	Adj. OR (95% CI)
Variable	N	%	n	%		
Total	70	100	140	100		
**Educational status**						
Illiterate	21	30	42	30	1.0(0.5–2.1)	-
Literate	49	70	98	70	Ref	
**Occupation**						
Daily wage labourer/ Agriculture cultivators	32	46	65	46	1.0(0.5–2.4)	-
Salaried	23	33	43	31	1.1(0.5–2.5)	
Unemployed	15	21	32	23	Ref	
**Marital Status**						
Ever Married	55	79	112	80	0.8(0.3–2.3)	-
Unmarried	15	21	28	20	Ref	
**Living locality**						
Urban	43	61	75	54	1.6(0.8–3.2)	-
Rural	27	39	65	46	Ref	
**History of household member with leprosy**						
Yes	12	17	18	13	1.4(0.6–2.9)	-
No	58	83	72	87	Ref	
**Alcohol consumption**						
Yes	33	47	43	31	2.4(1.2–4.8)*	2.3(1.1–4.9)**
No	37	53	97	69	Ref	Ref
**Messages related to leprosy program**						
Not Seen/heard/read	37	53	65	46	1.3(0.7–2.3)	-
Seen/heard/read	33	47	75	54	Ref	
**Health care provider met the patient in any survey**						
No	33	47	62	44	1.2(0.6–2.4)	-
Yes	37	53	78	56	Ref	
**Patient delay**						
More than 1 month	60	86	87	62	3.4(1.6–7.3)*	3.3(1.5–7.4)**
Less than or equal to 1 month	10	14	53	38	Ref	
**First HCP met**						
Private health care provider	48	69	65	46	2.6(1.4–4.9)*	2.6(1.4–5.2)**
Public health system	22	31	75	54	Ref	
**HCP delay**						
More than 2 week’s	55	79	93	66	2.1(1.01–4.5)*	-
Less than 2 week’s	15	21	47	34	Ref	
**Distance to nearest public health facility**						
More than 5 kms	28	40	51	36	1.2(0.6–2.1)	-
Less than or equal to 5 kms	42	60	89	64	Ref	

HCP-Health care provider;

* factors significant in univariate analysis with p-value <0.2,

** p-value < 0.05

Table 3 presents the reasons for not seeking health care immediately after the notice of 1^st^ symptom. Fifty three (79%) out of 67 patients with grade 2 deformity thought the symptom noticed was not a disease and it would disappear by itself and was the reason for not seeking health care immediately. Financial issues were stated as next major reason for not seeking health care immediately. Fifty four percent patients first visited a private health care provider.

**Table 3:** Reasons for not seeking care immediately after notice of first symptom among adult new leprosy patients with grade 2 disability at diagnosis registered in Maharashtra, India (2013–15).

Reasons for patient delay^#^	n 67*	% 100
Thought it was not a disease and would disappear by itself	53	79
No money to seek treatment	22	33
Due to family commitments	5	8
Health facility is far away	5	8

* Out of 70 cases, three sought care within a week thus they were excluded from this analysis

^#^ Multiple responses

Table 4 presents the reasons for not consulting doctor at the public health facility immediately after the notice of 1^st^ symptom. Fourteen (29%) out of 48 cases with grade 2 disability and 28 (43%) out of 65 controls with grade 0 disability perceived that long waiting time/unsuitable OPD timings was the reason for not seeking public health facility immediately. Fear of losing wages was stated as next major reason for not seeking health from public health facility immediately. Distance was reported as next major reason by controls and stigma of others will know the disease was stated as a reason by cases.

**Table 4:** Reasons for not consulting doctor at a public health facility immediately after notice of first symptom among new leprosy patients (grade 2 and grade 0 disability at diagnosis) registered in Maharashtra, India (2013–15).

Reasons for not consulting public health facility as an initial consultation*	Cases n = 48 (%)	Controls n = 65 (%)
Long waiting time/unsuitable OPD timings	14 (29%)	28 (43%)
Fear of losing wages	11 (23%)	20 (31%)
Government facility is far	8 (17%)	18 (28%)
Staffs are not approachable	9 (19%)	14 (22%)
Others will know my disease (Stigma)	7 (15%)	4 (6%)

* Out of 210 patients (70 cases and 140 controls) 113 consulted private provider first

### Qualitative findings

**Fig 2:**
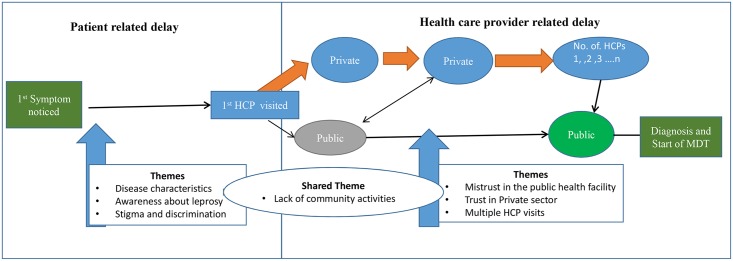
Patient pathway in Maharashtra, India (2013–15)—Themes associated with leprosy diagnosis delay.

Drawn from our qualitative data from patient and public health provider interviews, we constructed a “***care pathway***” model for patient with leprosy. The pathway was divided into two main parts; “***patient delays***” and “***health system delays***”. The emerging themes were organized along this model pathway and were related to its’ two distinct parts (Fig 2).

Individual pathways started from time of the first symptom or sign, which usually was a “skin lesion” with no significant other symptoms. After a period of “inaction” of variable duration the patient sought care either at the private or public health care facility. This was where the “health-system” part of the pathway started and it was completed when the patient was diagnosed with leprosy and put on treatment. As treatment was available mainly at the public health sector, the pathway for almost all patients ended at a public health care facility.

**Patient delays**:

“I just apply some extra powder to hide it”

Some clear themes emerged from the analysis of qualitative data analysis such as ‘the ***characteristics of the disease’***, especially the initial stages of the disease didn’t seem to alert patients and families. The lesions did not seem ‘threatening” nor “serious enough” to warrant medical care. Time and money to be spent to see a doctor were considered “a waste”. A young female leprosy patient from a semi-urban area close to Mumbai described her initial experiences with the disease:

“I got small round patch nearby elbow two years back. It was there for one year and I had no problem with the patch. So I did not see any doctor. Slowly that patch become little big, I got new soap and took bath two to three times daily. It was not troubling me so I did not go see any doctor.”

The narrative of a 40-year-old woman with grade 1 disability from the same geographical area is equally illustrative. One year back noticed *“… a small patch on my face*… *I applied cream but it did not disappear*, *after that I left caring for that patch*. *I just apply some extra powder to hide it*.*”*

All patients interviewed were unaware of the typical early manifestations of the disease. Patients had a variety of “self-diagnosis” from an “insect bite” to ‘allergy’ to ‘poisonous plants” to “ringworm”. The ***lack of awareness*** among the community in a high leprosy incidence setting was clear.

“Nobody in my area knows how leprosy attacks, very poor knowledge everybody has. I thought it was because of mosquito…”

(Male, 58 y, grade 2 disability)

In some cases, patients ***blamed themselves*** or felt that it was their fault that they delayed in seeking health care. The belief that the disease was ***“fate” or ‘God’s punishment”*** did not emerged prominently during the interviews apart from a 48-year-old man with grade 2 disease who clearly believed that the disease was “punishment”. Nevertheless. our interview schedule was not specifically targeted to explore deeper and more grounded beliefs around the disease.

The same themes about the nature and the characteristics of the disease at the early stages and the level of awareness among the community emerged from the health care provider interview data. According to a health care provider who has worked in the leprosy control programme for almost 15 years:

“People are not aware about leprosy and have less knowledge about its symptoms. People say that patches disappear on their own. They say it would be either ring worm or allergy; hence, they delay seeking health care…”

***Stigma and discrimination*** did not emerge as a main theme and reason for delay during the interviews with the patients. However, there were two cases of leprosy whose diagnosis was made early in the pathways of the patients but ignored the diagnosis and visited several more specialists (dermatologist, ophthalmologist, neurologist, orthopedician, etc) in private health care sector. This obvious ‘denial’ might imply stigma and discrimination however we haven’t specifically tried to elicit more in-depth accounts of stigma among the patients we interviewed. However, among health care providers this theme emerged strongly and was often associated with delays in seeking health care, especially among women, as often stigma was mentioned in the same context with marriage, divorce and uneducated rural populations:

“Sometime patients do not come to health facilities due to stigma, that’s a reason for delay too.”

(Health care provider, Mumbai, male 56 y)

“[there are] no cultural beliefs and traditional beliefs regarding leprosy disease in Mumbai but in rural area traditional beliefs like stigma is present so people sometimes hide their disease and some live separately.”

(Health care provider, Mumbai, male 48y).

An important theme arising again mostly from the providers’ interview data was the ***lack of community activities*** for leprosy, which were suspended after leprosy was declared ‘eliminated’ or ‘controlled’ by the central government. This important theme, even though is health-system related its’ main effect seemed to be on the levels of awareness in the community as well as the active case finding activities that would have significantly decreased delays in seeking health care. A leprosy doctor and official with almost 25 years of experience described clearly the difference in such activities:

“…Previously they had implemented SET pattern (survey, education, and treatment). More benefit for us, but now they do only survey. They are doing survey every year in government school but most children go to private school. It requires health education in high school and college student, because of this some children are missed out…”

**Health system delays**:

“Government doctors should treat us with compassion…”

An important “node” in the pathway model was identified at the point that the leprosy patient sought care for the first time for his/her disease. The vast majority of patients first visited a private physician at a nearby practice or facility. While the ***public health system*** was well equipped and resourceful to diagnose and treat leprosy, the first point of call for almost all patients was the ***private sector***, which in fact was not suitable to manage the disease and resulted in several circles of unnecessary investigations, misdiagnoses and several courses of inappropriate and therefore ineffective treatment.

There emerged a ‘deep-rooted’ belief that the government hospitals were not suitable but for very few cases and “only when the treatment was free”. Several reasons for this ***mistrust of the public health facilities*** and the staff were quoted such as; “lack of compassion among doctors”, “they don’t treat us good in government hospital”, “long waiting”, “I don’t like to go to PHC” and “doctors should be kind to patients”. One woman, 30 years old with grade 1 disability, described that her family goes usually to government hospital *“for all health problems”*. However, without any probing she added: *“We go early morning so that we get token and see doctor quickly”* indirectly pointing out to the main issues with government hospitals such as long waiting time and erratic presence of the staff.

Most people do not use negative expressions to describe their overall opinion with private doctors and facilities, despite their bad experience with delayed diagnosis and treatment of leprosy. They express frustration and regret in several circumstances:

*“I didn’t get time to go to doctor immediately*, *but when I went with small problem these doctors did so many tests and gave high dose medicine*. *So*, *I went to another doctor*. *Because of this my problem became severe*. *Doctor should tell correctly what the problem is without wasting our money [mala lagechch doctorankade janyasathi vel nahi milala pan jevha me kahi chhotya samasyansathi doctoranna bhetalo tevha tyanni khup vegvegalya tapasanya kela aani khup uchha doses chi aaushdhe dili*. *Mhunun me dusarya doctorankde gelo tyamule mazya samasya ajun vadhlya*. *Doctoranni aamacha paisa vaya n ghalavata*, *khar khar sangayala hav].”*

*(Male*, *58y*, *grade 2 disability)*

“I think; I did not give importance for that small skin patch in initial stage. I wasted 3 years, thinking it’s not a problem. After that only problems started, but doctors also didn’t tell correctly to me. I think time got wasted because of this.”

(Male, 60y, grade 2 disability)

However, while their mistrust of the public health sector seems deeply-rooted, they still largely ***trust the private sector*** and isolate the specific bad experience. A 48 years old male with grade 2 disability from District Thane opined, that Doctors should actually refer patients’ to the public health care facility where treatment is provided free of cost; *“Doctors should refer us to correct facility for free treatment [doctoranni aamha rugnanna yogya velet yogya rugnalayat pathavayala have]*.*”* The interview data revealed that the public sector was only accepted as a final, last resort, and after they were told that there were no more choices for correct diagnosis and treatment. The fact that both diagnosis and treatment at the government hospitals were free was appreciated by patients, but didn’t seem to alter significantly the overall, generalized negative belief towards the public and positive attitude towards the private health sector.

However, patients and health providers seemed to value the importance of the **community volunteers** of public health sector. Especially the role of Accredited Social Health Activists (ASHAs) in early identification and referral of patients to health facility was recognised. A-48-year old male described “….*When I went to my dad’s place ASHA met me in my home*, *and asked me to come to government hospital*. *I went to government hospital; doctor saw my walking pattern he told to use soft sole chappal*. *He told my son that I have leprosy and medicine should be taken for one year without stopping”*.

The proposed solutions, drawn from providers’ and patients’ interview, which were organized by the part of the model pathway they relate to, are shown in Table 5

**Table 5:** Solutions proposed by patients and health care providers to reduce diagnosis delay among adult new leprosy patients in Maharashtra, India (2013–15).

Categories	Codes/Themes	Verbatim quotes
**Patient related delay**	IEC programme	*‘Government should improve IEC material because this IEC material is very old’*.*–*(Provider)
		*‘In Tribal area there are local beliefs about the disease*, *they don’t seek care immediately*. *Traditional healers are the predominant treatment providers’*.*–*(Provider)
	Create awareness at patient level, community level and health care provider level	*‘There is less awareness about this disease*, *government should advertise this*, *if hospital staffs visit other offices*, *companies*, *industrial area*, *colleges more people will be more benefitted’*.*–*(Provider)
		*‘If they were informed about the sign and symptoms so we can prevent leprosy disease’*.*–*(Patient)
		*‘Leprosy should be included in text books about the disease*, *symptoms*, *and cure disease*. *It is required that teachers and paramedical workers*, *both*, *are trained for increasing awareness of leprosy among them and obtain their help in finding new cases’*.*–*(Provider)
**Health care Provider related delay**	Capacity building of HCPs	*‘…*.*doctors do not check nerves’–*(Provider)
		*‘Training required for every private medical practitioner’–*(Provider)
	Decentralisation and strengthening existing Public health facility	*‘Government say it is integrated programme but staffs are not actively involved in NLEP’–*(Provider)
	Increase community activities	*‘The leprosy cases will get detected early if household survey is done regularly’–*(Provider)

## Discussion

Our study revealed that patient and health system delays were significantly longer among leprosy cases with grade 2 disability compared to cases with grade 0 disability at diagnosis. The major contributor of the overall diagnosis delay was patient delay. Patient delay of more than one month was a risk factor for grade 2 disability. Many cases first met a private health care provider and this contributed to delayed diagnosis and appropriate management. The qualitative findings confirmed and added to our quantitative results.

Median total delay found in this study was 17.9 months (mean total delay was 23 months) among grade 2 cases and it was similar to a study in West Bengal which reported a mean total delay of 19.6 months [15]. It was lesser than the mean total delay of 37 months among grade 2 disability cases which was reported by a study conducted in Purulia [16]. A study in a South Indian at a tertiary care hospital revealed that mean patient delay was 7.9 months [17].

Ignorance of symptoms by patients and belief that symptom would disappear as reasons for not seeking health care have been also reported in other studies from India and other countries [15, 16, 17, 18, 19]. Waiting for the symptoms to disappear contributes for the longer patient delay. Qualitative data showed that stigma and discrimination might have an effect on health seeking behaviour however these issues were mostly highlighted by health providers and leprosy programme staff rather than by the leprosy patients. Stigma however has been consistently reported as a major issue with leprosy across different cultural settings throughout the human history [20].

‘In the present study, ‘long waiting time/unsuitable OPD timings’, ‘fear of losing wages’, ‘distance to public health care facility’, ‘non-approachability’ and ‘bad attitude of the hospital staff’ were the main reasons for avoiding the public health facilities. A study on undetected leprosy patients in Maharashtra had also reported that only few approach a government run health care facilities as a first visit to seek help [21]. There was trust in the private health care providers that contributed to the delay and mistrust in public health system that provided free leprosy treatment.

There is an increasing trend in grade 2 disability among new cases in the past 5 years [8]. After the declaration of leprosy elimination as a public health problem, funding for the leprosy program had reduced considerably [22]. In case of leprosy, elimination efforts were directed to control the disease burden rather than infection (by using prevalence instead of incidence of disease). Further, it appears that people, funders and health planners have equated ‘elimination of leprosy as public health problem’ i.e. prevalence of < 1 case per 10 000 population to elimination of a disease i.e. “reduction to zero of the incidence of a specified disease in a defined geographical area " [4].

The key recommendations that emerged from the analysis of the qualitative data are as follows: 1) increase awareness about leprosy and leprosy symptoms (including sensory symptoms) through Information Education Communication activities targeting patients, communities and health care providers 2) engagement of private practitioners in early referral to public health facility with special focus on specialists who are more likely to first see a patient with leprosy (dermatologists, neurologists, orthopaedics) 3) strengthen the general health system staff capacity in the early diagnosis of leprosy and involvement in field activities related to leprosy 4) active case finding as a strategy especially to target hot-spots in the district and 5) set district or community-specific targets for step by step elimination of the disease. “These recommendations if targeted at those “hot-spot” districts that have high proportion of grade 2 disability cases amongst new cases could help in decreasing the delay in seeking health care services from appropriate institutions.”

A major strength of this study was that it employed a mixed method design which proved invaluable in complementing and confirming quantitative and qualitative findings. The quantitative component was a matched case control study, which is a robust design for rare events. The fact that our data were ‘triangulated’ and we have adhered to the STROBE and COREQ guidelines added to the study strengths [13, 14]. The study had several limitations as well, first we did not ask questions about local attitudes and beliefs towards leprosy and we did not explore in-depth health-seeking behavior among the interviewees as well as stigma and discrimination experiences. This might have limited the richness of our qualitative data. However, as our study objectives were more descriptive and programmatic we focused mostly on the direct, operational reasons for delays. Further anthropological research may explore reasons for delay in presentation to appropriate health care facility where appropriate treatment is available and inform policies and strategies for the final steps of leprosy elimination. Private provider’s perceptions were not captured. Recall bias during patient interviews could not be avoided due to study design and no audio recording done during the interviews.

In conclusion, this study explored risk factors of grade 2 disability among new cases and perspectives of patients and health care providers. Promoting early case detection among new patients is a high priority given the neurological consequences of leprosy that occur in late diagnosis. The point of first contact with health care system (after a long or short patient delay) is a strong determinant for the early diagnosis and start of leprosy treatment. Hence strategy to actively engage private healthcare providers in National program is important for timely referral and early detection of leprosy cases and prevent disability.

## Supporting Information

S1 File Data set. (XLSX)

S2 File STROBE checklist. (PDF)
