# Supplementary material for: “I Wasted 3 Years, Thinking It’s Not a Problem”: Patient and Health System Delays in Diagnosis of Leprosy in India: A Mixed-Methods Study
Source: PLoS Negl Trop Dis. 2017 Jan 12;11(1):e0005192. doi: 10.1371/journal.pntd.0005192 (PMC5230757; doi:10.1371/journal.pntd.0005192)
Supplement: S2 File — (PDF) [file pntd.0005192.s002.pdf]

STROBE Statement—checklist of items that should be included in reports of observational studies

| | Item No. | Recommendation | Page No. | Relevant text from manuscript |
|---|---|---|---|---|
| **Title and abstract** | 1 | (*a*) Indicate the study's design with a commonly used term in the title or the abstract | 2 | Included in methods section |
| | | (*b*) Provide in the abstract an informative and balanced summary of what was done and what was found | 2 | Included in abstract |
| **Introduction** | | | | |
| Background/rationale | 2 | Explain the scientific background and rationale for the investigation being reported | 5 | Included in Introduction |
| Objectives | 3 | State specific objectives, including any prespecified hypotheses | 5 | Included in Introduction |
| **Methods** | | | | |
| Study design | 4 | Present key elements of study design early in the paper | 6 | Included in study design |
| Setting | 5 | Describe the setting, locations, and relevant dates, including periods of recruitment, exposure, follow-up, and data collection | 6 &7 | Included in study setting |
| Participants | 6 | (*a*) *Case-control study*—Give the eligibility criteria, and the sources and methods of case ascertainment and control selection. Give the rationale for the choice of cases and controls | 7 | Included in study population |
| | | (*b) Case-control study*—For matched studies, give matching criteria and the number of controls per case | 7 | Included in study population and sample population |
| Variables | 7 | Clearly define all outcomes, exposures, predictors, potential confounders, and effect modifiers. Give diagnostic criteria, if applicable | 8 | Included in data collection and variables |
| Data sources/ measurement | 8* | For each variable of interest, give sources of data and details of methods of assessment (measurement). Describe comparability of assessment methods if there is more than one group | *9* | Included in data collection and variables |
| Bias | 9 | Describe any efforts to address potential sources of bias | - | - |
| Study size | 10 | Explain how the study size was arrived at | 7 | Included in sample size and sampling |

Continued on next page

| | | | | |
|---|---|---|---|---|
| Quantitative variables | 11 | Explain how quantitative variables were handled in the analyses. If applicable, describe which groupings were chosen and why | 8 | Included in data collection variables |
| Statistical methods | 12 | (*a*) Describe all statistical methods, including those used to control for confounding | 9 | Included in data analysis |
| | | (*b*) Describe any methods used to examine subgroups and interactions | 9 | Included in data analysis |
| | | (*c*) Explain how missing data were addressed | 9 | Included in data analysis |
| | | (*d*) *Case-control study*—If applicable, explain how matching of cases and controls was addressed | 9 | Included in data analysis |
| | | (*e*) Describe any sensitivity analyses | - | - |
| **Results** | | | | |
| Participants | 13* | (a) Report numbers of individuals at each stage of study—eg numbers potentially eligible, examined for eligibility, confirmed eligible, included in the study, completing follow-up, and analysed | 10 | Included in Results |
| | | (b) Give reasons for non-participation at each stage | - | - |
| | | (c) Consider use of a flow diagram | - | - |
| Descriptive data | 14* | (a) Give characteristics of study participants (eg demographic, clinical, social) and information on exposures and potential confounders | 10 & 11 | Included in Results |
| | | (b) Indicate number of participants with missing data for each variable of interest | - | - |
| Outcome data | 15* | *Case-control study*—Report numbers in each exposure category, or summary measures of exposure | 10 & 11 | Included in Results |
| Main results | 16 | (*a*) Give unadjusted estimates and, if applicable, confounder-adjusted estimates and their precision (eg, 95% confidence interval). Make clear which confounders were adjusted for and why they were included | 10 & 11 | Included in Results |
| | | (*b*) Report category boundaries when continuous variables were categorized | 10 & 11 | Included in Results |
| | | (*c*) If relevant, consider translating estimates of relative risk into absolute risk for a meaningful time period | 10 & 11 | Included in Results |

Continued on next page

| | | | | |
|---|---|---|---|---|
| Other analyses | 17 | Report other analyses done—eg analyses of subgroups and interactions, and sensitivity analyses | 12 & 13 | Table 3 & 4 |
| **Discussion** | | | | |
| Key results | 18 | Summarise key results with reference to study objectives | 20 | Included in discussion |
| Limitations | 19 | Discuss limitations of the study, taking into account sources of potential bias or imprecision. Discuss both direction and magnitude of any potential bias | 21 | Included in discussion |
| Interpretation | 20 | Give a cautious overall interpretation of results considering objectives, limitations, multiplicity of analyses, results from similar studies, and other relevant evidence | 21 & 22 | Included in discussion |
| Generalisability | 21 | Discuss the generalisability (external validity) of the study results | 21 & 22 | Included in discussion |
| **Other information** | | | | |
| Funding | 22 | Give the source of funding and the role of the funders for the present study and, if applicable, for the original study on which the present article is based | 23 | Included in Acknowledgement |
